# *Anaplasma marginale* and *A. phagocytophilum* in cattle in Tunisia

**DOI:** 10.1186/s13071-016-1840-7

**Published:** 2016-10-20

**Authors:** Youmna M’ghirbi, Marwa Bèji, Beatriz Oporto, Fatma Khrouf, Ana Hurtado, Ali Bouattour

**Affiliations:** 1 Université Tunis El Manar, Institut Pasteur de Tunis, Laboratoire d’Epidémiologie et de Microbiologie Vétérinaire, Service d’Entomologie Médicale, 1002 Tunis-Belvédère, Tunisia; 2 Department of Animal Health, NEIKER – Instituto Vasco de Investigación y Desarrollo Agrario, Berreaga 1, 48160 Derio, Bizkaia Spain

**Keywords:** *Anaplasma marginale*, *Anaplasma phagocytophilum*, Cattle, Duplex PCR assay, Tunisia

## Abstract

**Background:**

Tick-borne diseases caused by *Anaplasma* species put serious constraints on the health and production of domestic cattle in tropical and sub-tropical regions. After recovering from a primary infection, cattle typically become persistent carriers of pathogens and play a critical role in the epidemiology of the disease, acting as reservoirs of the *Anaplasma* spp.

**Methods:**

In this study a duplex PCR assay was used for the simultaneous detection of *Anaplasma marginale* and *Anaplasma phagocytophilum* in cattle using two primer pairs targeting *msp*4 and *msp*2 genes, respectively. We used this method to analyze DNA preparations derived from 328 blood cattle samples that were collected from 80 farms distributed among Tunisia’s four bioclimatic zones.

**Results:**

The prevalence of the *A. marginale* infection (24.7 %) was significantly higher and more widespread (in all bioclimatic areas) than that of *A. phagocytophilum* (0.6 %), which was found in a mixed infection with *A. marginale*.

**Conclusions:**

The duplex PCR assay used proved to be a rapid, specific and inexpensive mean for the simultaneous detection of *Anaplasma marginale* and *Anaplasma phagocytophilum* in cattle blood. It allowed us to report the identification of *A. phagocytophilum* for the first time in cattle in Tunisia and confirm the presence of *A. marginale* in cattle from several geographical areas of the country. Further epidemiological studies undertaken using this assay will help improve the surveillance of the associated diseases in the regions where they are endemic.

## Background

Among tick-borne diseases, bovine anaplasmosis is considered to be one of the most important in ruminants worldwide, causing significant economic losses in tropical and subtropical areas [1]. The socioeconomic impact of the disease and the restrictions on trading infected animals internationally led the Office International des Epizooties (OIE) Animal Health Code to categorize anaplasmosis as a disease that required a notification of its presence [2]. Because outbreaks are seasonal and infection rates are stable, the significance of anaplasmosis is underestimated in endemic areas [3]. Cattle can be infected by several *Anaplasma* species, like *A. marginale*, *A. phagocytophilum*, *A. centrale* and *A. bovis* [4–6]. *Anaplasma marginale* is one of the most prevalent tick-transmitted rickettsial diseases of cattle in the world [7]. Highly pathogenic, especially in cattle up to two years old, it causes a disease that produces progressive anemia and icterus [8]. Several decades ago *A. phagocytophilum* (formerly known as *Ehrlichia phagocytophila*, *E. equi* and human granulocytic ehrlichiosis agent), was identified in cattle; it may also infect humans [9]. Known to cause tick-borne fever in cattle, it causes not only high fever, but also coughs, miscarriages, decreased milk production and loss of appetite [10]. In areas infested by several tick vector species and where animal husbandry practices include vaccination with live *A. centrale* bacteria (Israel, Africa, Australia and parts of South America), cattle can be co-infected with two or more *Anaplasma* species [11, 12]. Disease treatment and prevention strategies focus on using reliable diagnostic tests to accurately and precisely identify infected cattle. While inoculating splenectomized cattle with whole blood has been the gold standard for determining persistent *A. marginale* infections in cattle, it is not required for routine testing [13]. Bovine anaplasmosis is diagnosed by identifying *Anaplasma* in Giemsa-stained blood smears from clinically suspect animals during the acute phase of the disease. However, this method is not useful for detecting pre-symptomatic and carrier animals. Currently, the competitive enzyme-linked immunosorbent assay (cELISA) is one of the most common diagnostic techniques used to identify the bovine anti-major surface protein 5 (anti-MSP5) of *Anaplasma marginale* [14]. It is considered to be a reliable screening test for cattle infected with *A. marginale* and to establish their carrier state. However, cross-reactivity has been reported when the cELISA is used to classify cattle infected with *A. marginale* and/or *A. phagocytophilum* [15, 16]. Several other serological tests have been used extensively in epidemiological studies of anaplasmosis despite the fact that they do not discriminate between different, antigenically similar *Anaplasma* species [16, 17]. Yet highly sensitive and specific, molecular methods have been developed to identify *A. marginale* and *A. phagocytophilum* DNA [18–22]. To develop a robust diagnostic method, an appropriate target needs to be selected in order to accurately and precisely determine an infection.

In Tunisia, Rickettsiales species including *A. phagocytophilum*, *A. bovis*, *A. marginale*, *A. centrale*, *Ehrlichia canis*, *Ehrlichia* sp. and *A. platys* have recently been detected in horses, cattle, small ruminants, camels, dogs and ticks [23–29]. A molecular assay based on a single-step duplex PCR, was used to simultaneously detect and differentiate *A. marginale* and *A. phagocytophilum* and determine their distribution in cattle from Tunisia.

## Methods

### Design of primers

*A. marginale msp*4 gene sequences and *A. phagocytophilum msp*2 gene sequences were aligned with those of other related species of the genera *Anaplasma* and *Ehrlichia* using Vector NTI 8.0 software (Informax Inc., North Bethesda, MD, US). Primers (Table 1) were designed to specifically amplify a 420 bp fragment of the *msp*4 gene of *A. marginale* and used in combination with the previously designed primer pair to amplify a 334 bp fragment of the *msp*2 gene of *A. phagocytophilum* [30].

**Table 1:** Primers used in this study

Species	Target gene	Primer	Sequence 5’–3’	Reference
*A. marginale*	*msp*4	M4-OvMar-F	ATCTTTCGACGGCGCTGTG	This study
		M4-Mar-R	ATGTCCTTGTAAGACTCATCAAATAGC	
*A. phagocytophilum*	*msp*2	Msp2-3 F	CCAGCGTTTAGCAAGATAAGAG	[30]
		Msp2-3R	GCCCAGTAACAACATCATAAGC	

### Cloning and sequencing the *msp*4 *A. marginale* gene and *msp*2 *A. phagocytophilum* gene

DNA was extracted from whole blood samples of two cows naturally infected with *A. marginale* and *A. phagocytophilum* using QIAamp DNA Mini Kit (QIAGEN, Hilden, Germany) as per the manufacturer’s recommendations, and extracted DNA was used as template to amplify a 420 bp (*msp*4 gene) and 334 bp (*msp*2 gene), respectively. The amplified products were cloned into a pCR4-TOPO vector and introduced into chemically competent *Escherichia coli* as per the manufacturer's instructions (TOPO TA cloning kit for sequencing; Invitrogen, Carlsbad, California). Recombinant plasmid DNA was purified using a FlexiPrep kit (Amersham Biosciences, Freiburg, Germany) and subjected to automatic dye terminator cycle sequencing. The nucleotide sequences of the plasmid inserts were confirmed as *A. marginale* and *A. phagocytophilum* by comparing them with the GenBank database.

The concentration of each plasmid was calculated with a NanoDrop® ND-1000 (Thermo Scientific, Wilmington, DE, USA) spectrophotometer and the plasmids were 10-fold serially diluted in a Tris-EDTA buffer to reach concentrations ranging from 10^8^ to 10 copies/μl. Serial dilutions of individual plasmids as well as different combinations were tested to calculate the sensitivity of the assay.

### Duplex amplification

PCR reactions were performed using a commercially available Multiplex-PCR assay kit (QIAGEN, Hilden, Germany) in 25 μl volume reactions that include 1× QIAGEN Multiplex PCR Master Mix (QIAGEN), 0.5 μM of Msp2-3 F/Msp2-3R primers, 0.2 μM M4-OvMar-F/M4-Mar-R primers and 5 μl of extracted DNA. Cycling conditions were 15 min at 95 °C, followed by 40 cycles of 94 °C for 30 s, 63 °C for 90 s, 72 °C for 90 s and 72 °C for 10 min. To avoid cross-contamination and false-positive reactions, we used plugged tips, set PCRs in separate rooms, and also included a negative (water) control in each run.

### Sensitivity and specificity of single and duplex PCR assay

To determine the detection limit of single and duplex PCRs, 10-fold serial dilutions of individual plasmids with the insert of *A. phagocytophilum* and *A. marginale* as well as different combinations were tested under the conditions described above. Sensitivity was also tested on DNA extracted from blood from a non-infected cow spiked with these same plasmid combinations (Table 2). Specificity was tested using DNA from other species (*Anaplasma ovis*, *Anaplasma platys*, *Ehrlichia* sp., *Ehrichia canis* and *Rickettsia conorii*).

**Table 2:** Analytical sensitivity of the duplex PCR assay

Plasmid copies^a^	DNA uninfected cattle^b^	*A. phagocytophilum*	*A. marginale*
10 AP	N	Positive	Negative
10 AP	Y	Positive	Negative
1 AP	N	Negative	Negative
1 AP	Y	Negative	Negative
10 AM	N	Negative	Positive
1 AM	N	Negative	Positive
1 AM	Y	Negative	Positive
10^3^ AP + 10 AM	N	Positive	Positive
10^3^ AP + 10 AM	Y	Positive	Positive
10 AP + 10^3^ AM	N	Positive	Positive
10 AP + 10^3^ AM	Y	Positive	Positive

^a^AP, plasmid with an insert of the *msp*2 gene fragment of *Anaplasma phagocytophilum*; AM, plasmid with an insert of the *msp*4 gene fragment of *Anaplasma marginale*

^b^Presence (Y) or absence (N) in the PCR reaction of DNA extracted from blood from a non-infected cow spiked with the indicated plasmid or plasmid combinations

### Study design and sampling approach

**Fig. 1:**
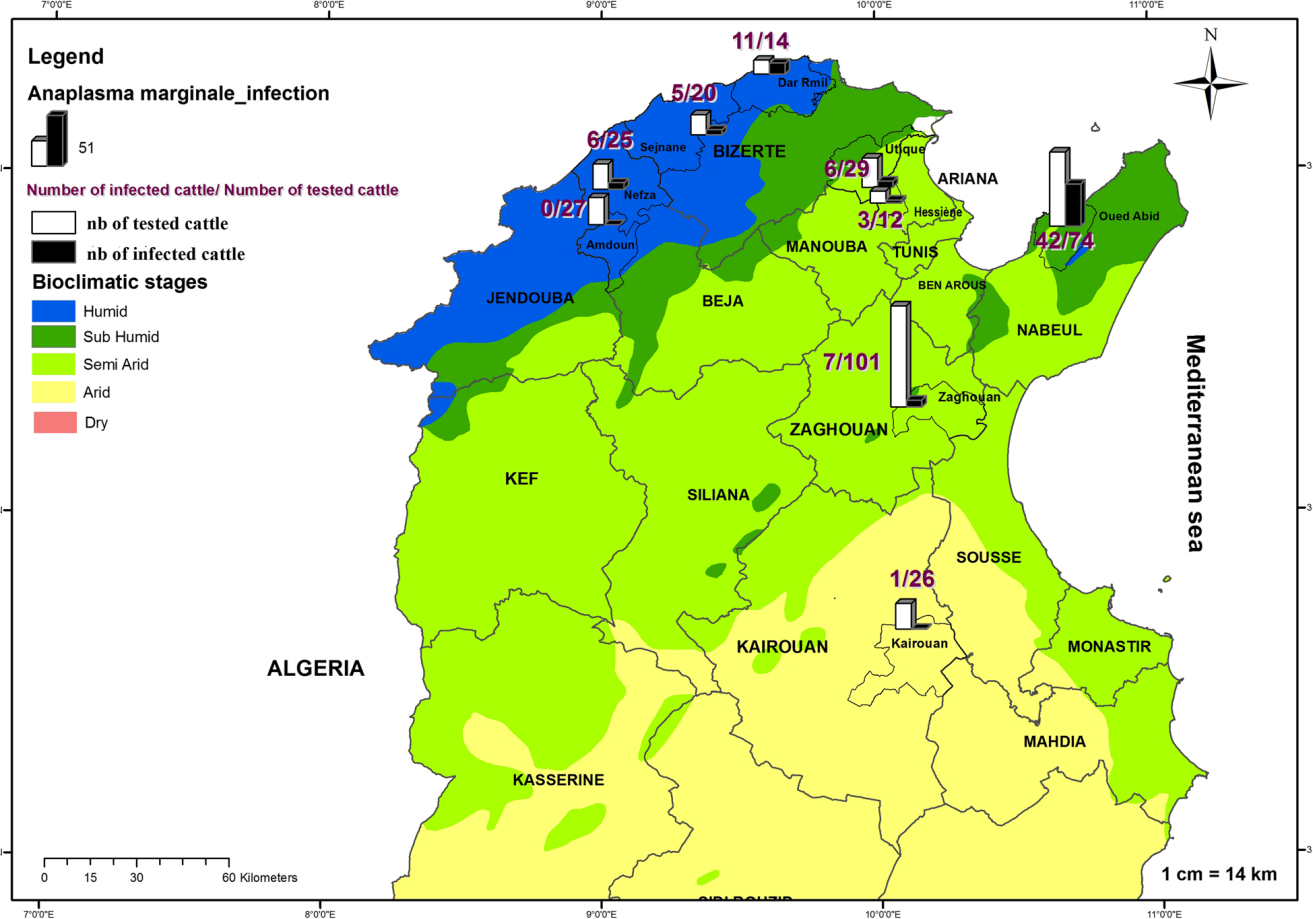
Map of Tunisia showing the different studied localities with the number of tested and infected cattle by *Anaplasma marginale*

A cross-sectional study was carried out in 9 localities, located in 4 different bioclimatic zones, in northern and central Tunisia (humid, sub-humid, semi-arid and arid) where cattle’s breeding is an important economic activity (Fig. 1). All localities have a Mediterranean climate - cool, moist winters and dry, hot summers. Topographically, the areas have rolling hills interspersed with farmland, grassland, oak woodlands and Mediterranean scrub (*Olea europaea*, *Pistacia lentiscus*, *Cistus monspeliensis,* etc.). A total of 80 farms with fewer than 30 animals per farm were chosen randomly as representative of the local management system on the basis of the recommendations of the State Veterinary Office. Animal husbandry practices are generally traditional small herds grazing on permanent pastures or bush. A total of 328 cattle were sampled of which 37.2 % were local breed, 32.3 % cross-breeds, 18 % Friesian, 9.2 % Schwytz and 3.4 % Holstein. Animals ranged in age between 3 months and 13 years, and most were dairy cattle (97.6 %).

### Blood sample collection and DNA extraction

Animals were bled once between June and November, a period during which they are typically grazing in pastures and exposed to tick bites. Blood was sampled in tubes containing ethylenediamine tetraacetic acid and DNA was extracted using the QIAamp DNA Mini Kit (QIAGEN, Hilden, Germany). DNA yields were determined with a Nano-Drop® ND-1000 Spectrophotometer (Nano-Drop Technologies, DE, USA).

DNA samples were subjected to duplex PCR assay in order to detect *A. marginale* and *A. phagocytophilum* as described above, and amplicons were resolved in ethidium bromide-stained agarose (Gellyphor, EuroClone, Milan, Italy) gels (1.5 %) and measured by comparing them with the with Gene RulerTM 100-bp DNA Ladder (MBI Fermentas, Vilnius, Lithuania) as molecular marker. Gels were photographed using Gel Doc 2000 (Bio-Rad, Hercules, CA, USA).

### Sequencing and data analysis

The specificity of the duplex PCR was confirmed by sequencing PCR amplicons of *A. marginale* and *A. phagocytophilum* using primers M4-OvMar-F/M4-Mar-R for *msp*4 gene and Msp2-3 F/Msp2-3R for *msp*2, respectively. Thirteen randomly chosen positive PCR products were purified using the ExoSAP cleanup procedure (Amersham Biosciences, Piscataway, NJ, USA). All nucleotide sequences were obtained using the Big Dye Terminator v.3.1 Cycle Sequencing Kit (Applied Biosystems, Foster City, CA, USA) and the 3130 automated sequencer (Applied Biosystems). The sequences were edited and aligned using DNA Baser Sequence Aligner v3.5.4 software (Heracle BioSoft SRL, www.DnaBaser.com) to obtain optimal sequence alignment files. A BLAST analysis was made in the NCBI database to retrieve sets of homologues exhibiting high scores with the partial *msp*2 and *msp*4 gene of *A. phagocytophilum* and *A. marginale*, respectively.

### Statistical analysis

The Chi-square or Fisher's exact tests were used to compare proportions of positivity in relation with bioclimatic zone, breed and sex. Observed differences were considered significant when the resulting *P*-value was less than 0.05.

### Nucleotide sequence accession numbers

Sequence data were deposited in GenBank; accession numbers for the partial *msp*2 and *msp*4 sequences are KR871275–KR871287.

## Results

### Performance of the duplex PCR assay

Fragments of the expected size were generated from the template plasmids representing *A. marginale* (420 bp) and *A. phagocytophilum* (334 bp); while DNA from uninfected bovines used as negative control, displayed no evidence of fragment amplification. Similarly, no amplicons were obtained when testing DNA from other species (*Anaplasma ovis*, *A. platys*, *Ehrlichia* sp., *E. canis* and *Rickettsia conorii*). The PCR was able to detect 1 copy of *A. marginale* and 10 copies of *A. phagocytophilum* plasmid templates, when present as single infections, and 10 copies in mixed infections even when the differences in their concentrations were of two orders of magnitude and in the presence of uninfected host DNA (spiked controls).

### Analysis of blood samples by PCR duplex assay

A total of 83 cattle (representing 25.3 % of the analyzed animals) were infected with *A. marginale* and/or *A. phagocytophilum* (Table 3). In 24.7 % (81/328) of analyzed cattle *A. marginale msp*4 amplicons were detected and, in another 0.6 % (2/328) of the animals, PCR results confirmed the presence of *A. phagocytophilum* DNA as a mixed infection with *A. marginale* (Table 3). None were positive for only *A. phagocytophilum*. The two cases of mixed infections were identified in the humid and sub-humid zones (Table 3), while *A. marginale* was detected with different rates in humid (25.6 %), sub-humid (46.6 %), semi-arid (8.8 %) and arid zones (4 %); the difference being significant (*χ* ^2^ = 47.95, *df* = 3, *P* < 0.0001) (Table 3). Infection rates of *A. marginale* were statistically higher in Schwyz breed (56.7 %) than in other breeds (*χ* ^2^ = 32.2, *df* = 4, *P* < 0.0001). The lowest prevalence was observed in Black Friesians (15.2 %; Table 4). Cattle with a mixed infection were local and Friesian black breeds (Table 4). Proportion of animals infected with *A. marginale* was significantly different (*χ* ^2^ = 7.22, *df* = 1, *P* = 0.0072) in cattle younger (2/34; 5.9 %) and older than (79/294; 26.9 %) one year. The two co-infected cattle were older than one year of age.

**Table 3:** Duplex PCR detection and identification of *Anaplasma* species in cattle in the four studied bioclimatic zones

Bioclimatic zones	Localities	*n* cattle/*n* farms	*A. marginale* (%)	*A. phagocytophilum* (%)	*A. marg* + *A. phag* (%)
Humid	Sejnane	20/5	5 (25.0)	0	0
	Dar Rmil	14/3	11 (78.5)	0	0
	Nefza	25/3	6 (24.0)	0	1 (4.0)
	Amdoun	27/5	0	0	0
Total HUMID		86/16	22 (25.6)	0	1 (1.2)
Sub-humid	Utique	29/5	6 (20.7)	0	0
	Oued Abid	74/7	42 (56.7)	0	1 (1.4)
Total SUB-HUMID		103/12	48 (46.6)	0	1 (1.0)
Semi-arid	Zaghouan	101/44	7 (6.9)	0	0
	Hessiène	12/3	3 (25.0)	0	0
Total SEMI-ARID		113/47	10 (8.8)	0	0
Arid	Kairouan	26/5	1 (3.8)	0	0
Total ARID		26/5	1 (3.8)	0	0
Total		328/80	81 (24.7)	0	2 (0.6)

**Table 4:** Prevalence of cattle by breed infected with *A. marginale* and *A. phagocytophilum*

Breed (*n*)	*A. marginale* (%)	*A. phagocytophilum* (%)	*A. marg + A. phag* (%)^a^	Negative (%)	Total (%)
Local (122)	31 (25.4)	0	1 (0.8)	90 (73.8)	32 (26.2)
Cross-bred (106)	24 (22.7)	0	0	82 (77.4)	24 (22.6)
Friesian (59)	9 (15.3)	0	1 (1.7)	49 (83.1)	10 (17.0)
Schwytz (30)	17 (56.7)	0	0	13 (43.3)	17 (56.7)
Holstein (11)	0	0	0	11 (100.0)	0
Total (328)	81 (24.7)	0	2 (0.6)	245 (74.7)	83 (25.3)

^a^ *A. phagocytophylum* was always found as a mixed infection with *A. marginale*

### Sequence analyses

To confirm the PCR results, 11 PCR products positive for *A. marginale* (from 9 investigated localities) and two for *A. phagocytophilum* were sequenced. A BLAST analysis of the obtained sequences revealed genetic variability among *A. marginale* at five nucleotide positions (354, 423, 538, 564, 714) (Table 5). The 11 sequences (GenBank accession numbers KR871277–KR871287) showed significant identity (99–100 %) with *A. marginale* sequences described in Italy (GenBank accession number DQ000618), USA (GenBank accession number AY253143) and Spain (GenBank accession number AY456003). Four sequences (KR871279, KR871284, KR871281, KR871283) presented overlapping peaks at four positions. As they were confirmed by re-sequencing, they are indicative for double infections rather than for errors introduced during sequencing (Table 5). In addition, the two partial sequences of the *msp*2 gene of *A. phagocytophilum* (KR871275, KR871276) were identical between them and showed 100 % identity to sequences detected in white-tailed deer (DQ097228) and humans (AF135255) in America.

**Table 5:** *A. marginale* sequencing analysis results

GenBank accession number	Locality	Blast analysis	Similarity (%)	Host (Country)	Nucleotide positions^a^				
					354	423	538	564	714
KR871277	Dar Rmil	AY253143	100	Bison (USA)	A	G	T	A	C
KR871284	Nefza	AY253143	99	Bison (USA)	A	R	T	R	C
KR871280, KR871285 to KR871287	Oued Abid	DQ000618	100	Bison (Italy)	G	A	T	G	C
KR871282	Hessiène	DQ000618	100	Bison (Italy)	G	A	T	G	C
KR871279	Sejnane	DQ000618	99	Bison (Italy)	R	A	T	G	C
KR871278	Zaghouan	AY456003	100	Deer (Spain)	G	G	T	A	C
KR871281	Utique	AY456003	99	Deer (Spain)	R	G	T	A	C
KR871283	Kairouan	DQ000618	99	Cattle (Italy)	G	A	T	G	Y

*Abbreviations*: *R* degenerated nucleotide (A/G), *Y* degenerated nucleotide (C/T)

^a^Nucleotide positions are indicated referring to the complete *msp*4 gene sequence (e.g. AF428081)

## Discussion

Rickettsial (*Anaplasmataceae*) bacteria are recognized as emerging tick-borne pathogens that are important for humans and animals [31, 32]. Changes in the host-vector ecology are largely responsible for the emergence of these pathogens. Moreover, the new insights in the development of laboratory tools for the detection of *Anaplasma* infections have contributed to the detection of new species [33]. Indeed, the global threat of *Anaplasmataceae* highlights the need for new tools able to discriminate among the different species. Several molecular techniques were therefore proposed for detecting and characterizing species belonging to the family *Anaplasmataceae*. Most of these techniques target the major surface proteins (MSPs) [18], the heat-shock gene *groEL* [19], the 23S rRNA [20] and the 16S rRNA gene [21]. Here, we targeted the *msp*4 and *msp*2 genes, which are involved in host-pathogen and tick-pathogen interactions and have been used as markers for the genetic characterization of *A. marginale* strains [18], using a conventional PCR for the detection of *A. phagocytophilum* and *A. marginale* in a duplex format. The optimized duplex PCR was able to specifically detect *A. phagocytophilum* and *A. marginale* from both single and mixed DNA preparations without affecting the detection limit. The field samples provided further evidence of the assay’s applicability. Indeed, only amplicons of the expected size were generated and the results of the duplex PCR entirely corresponded with the results obtained by sequencing the amplicons generated. This duplex PCR allowed us to report the identification of *A. phagocytophilum* for the first time in cattle in Tunisia and confirm the presence of *A. marginale* in cattle from several geographical areas of the country.

The obtained prevalence of *A. marginale* (24.7 %) was lower than that reported in Kansas (37.6 %) [21], India (73.1–36.8 %) [34, 35], Sicily (50 %) [36], Brazil (70.2 %) [37], South African provinces (65–90 %) [38], Texas (82 %) [39] and Costa Rica (56.9 %) [40]. By contrast, this prevalence was higher than those recorded in Turkey (2.8 %) [41], Egypt (21.3 %) [42] and the Philippines (19.8 %) [43]. The significant prevalence of *A. marginale* warrants further investigation to evaluate the impact of this bacterium on livestock production, since it is considered to be a pathogenic species in cattle in North Africa, causing severe clinical symptoms and very serious economic losses [44]. However, at the time of blood sampling (June-November), the 83 cattle infected with *A. marginale* showed no clinical signs. These animals could be considered asymptomatic carriers. Indeed, Sergent et al. [44] have shown that North African strains of *A. marginale* confer an immune protection in experimentally infected animals.

*Anaplasma marginale* was identified in all the bioclimatic zones where we carried out our investigations, however, its prevalence was highest in farms in the sub-humid zone (46.6 %) compared to those in the humid (25.6 %), semi-arid (8.8 %) and arid (3.8 %) zones. These results concur with previous findings in Morocco [45], demonstrating a higher prevalence of infection with *A. marginale* in cattle in sub-humid (52 %) zones compared to humid (22.7 %) and semi-arid zone (20 %). The observed variations in the distribution of *A. marginale* in the different bioclimatic zones and farms in the same locality could be explained by the diversity of the Ixodidae fauna that parasitize cattle in each locality and farm. The difference can also be attributed to each farmer’s management of pasture livestock. In a given bioclimatic region, the latter factor is closely related to tick infestation of the cattle. These results were correlated with the presence of tick vectors of *A. marginale* and *A. phagocytophilum*, mainly *Hyalomma* spp., *Rhipicephalus* spp. and *Ixodes* spp. [46, 47].

A significant difference was observed in *A. marginale* infections between cattle breeds (*P* < 0.05), with the highest prevalence in the Schwytz breed. In Uganda, Magona and Mayende [48] reported a high rate of mortality in Friesian cattle due to a high prevalence of *A. marginale* associated or not with other pathogens (*Theileria* and *Trypanosoma*). Our results contradict those reported in Morocco by Ait Hamou et al. [45], where they described a non-significant difference in the prevalence of infection with *A. marginale* among cross, local and imported breeds. In fact, in North Africa, local and cross-breed animals are considered more resistant to anaplasmosis than pure breeds imported from Europe. But it seems that this relative resistance is due less to the breed than to the fact that animals born in endemic areas acquire a natural immunity (premunition) at an early age [44].

The age of cattle appears to influence the prevalence of anaplasmosis, *A. marginale* infection rate being significantly higher in older animals. Similarly, in Morocco, Ait Hamou et al. [45] reported the difference in the prevalence of *A. marginale* infections in calves (26.1 %) and adults (52.4 %). Our results were consistent with those reported in Uganda [48]. This difference might be explained by the more sustained exposure of adults to tick vectors [49]. Moreover, it appears that calves are less susceptible to the disease. Indeed, anaplasmosis is rare in animals younger than six months, while those between six months and one year usually develop only a mild illness, and cattle between one and two years old develop multiple signs of the disease, which is rarely fatal. However, the disease is often fatal after acute infection in adults over two years old, with a mortality risk ranging from 29 % to 49 % [7, 50]. Calves are temporarily protected (maternal antibodies) by the colostrum and a mother’s immunity, preventing short-term protection.

In our study, the prevalence of *A. phagocytophilum* (0.6 %) was much lower than that of *A. marginale*. This concurred with the results reported in Turkey where the prevalence of *A. marginale* (2.8 %) was higher than that of *A. phagocytophilum* (1 %) [41] but contradicted the study carried out in Italy by Torina et al. [51], where the prevalence of *A. phagocytophilum* (16.6 %) was higher than that of *A. marginale* (9.8 %). This difference may be attributed to (i) the fluctuation of the bacteraemia during the chronic phase of *A. phagocytophilum* and *A. marginale* infection [52]; (ii) the low number of intragranulocytic *A. phagocytophilum* circulating in carrier animals [53]; (iii) the fact that *A. marginale* develops faster than *A. phagocytophilum* in the host [54]; (iv) to the higher density of competent vectors and/or reservoirs; or (v) to the different tick infection rates by *A. marginale* and *A. phagocytophilum*.

Sequencing results showed a genetic variability in Tunisian *A. marginale* consistent with the great *A. marginale* genetic diversity found in endemic areas worldwide [18]. Presence of overlapped peaks at certain positions of four of the samples (sequences KR871279, KR871284, KR871281, KR871283) even after re-sequencing would suggest that certain animals harbored double infections, a circumstance already reported [55].

## Conclusions

In conclusion, the duplex assay used here offered a rapid, specific and inexpensive mean of discriminating between *A. marginale* and *A. phagocytophilum* in carrier cattle. We now use this system routinely in our research and have tested it on blood samples, ticks, and fleas (data not shown). This technique could be used to detect these pathogens in tick vectors whose activity and abundance are affected by global warming [56]. The present study also determined the prevalence of the two pathogens and identified different *A. marginale* variants in cattle from different areas in Tunisia. More epidemiological studies are needed to determine the vectors and reservoir animals for the *Anaplasma* species and to clarify the pathogenicity of *A. marginale* and *A. phagocytophilum* for humans and animals in Tunisia.

## Abbreviations

A: *Anaplasma*
BLAST: Basic local alignment search tool
cELISA: Competitive enzyme-linked immunosorbent assay
E: *Ehrlichia*
*groEL*: Heat-shock gene
*msp*2: Major surface protein 2 gene
*msp*4: Major surface protein 4 gene
MSP5: Major surface protein 5
MSP5: Major surface protein 5
MSPs: Major surface proteins
NCBI: National Center for Biotechnology Information
OIE: The Office International des Epizooties
